# Heat stress alleviation and dynamic temperature measurement for growing beef cattle

**DOI:** 10.1093/tas/txaa144

**Published:** 2020-12-22

**Authors:** Sheyenne M Augenstein, Meredith A Harrison, Sarah C Klopatek, James W Oltjen

**Affiliations:** Department of Animal Science, University of California Davis, Davis, CA

## INTRODUCTION

Heat stress has caused an estimated $370 million annually in economic losses for the beef industry (St-Pierre et al., 2003), pressuring producers to implement effective mitigation strategies. Common heat abatement strategies include providing shade or sprinkler systems, which both can be an effective method of cooling cattle (Mitlöhner et al., 2002; Kendall et al., 2007). However, only 13–14% of feedlots in the United States provide at least one method of cooling beef cattle during elevated heat conditions (USDA, 2000). Many feeder states are located in climates where heat abatement strategies are beneficial. Sprinkler systems can be a strategic management choice in California, the sixth largest feeder state, due to climate characteristics, such as high heat and low humidity.

With increased ambient temperature, the ability for beef cattle to dissipate body heat decreases, resulting in physiological and behavioral modifications that affect gain and carcass quality. Cattle may decrease their feed intake and feeding patterns may shift to cooler portions of the day or smaller, more frequent visits to the bunk (Hahn, 1999). In addition, cattle may seek shade or areas closer to water sources (Blackshaw and Blackshaw, 1994). Although research shows that sprinkler systems can be an effective heat abatement strategy in feedlots, the studies are generally implemented on a smaller scale under controlled conditions and do not consider variable environmental situations for sprinkler activation. Our study aims to evaluate the ability of sprinkler systems to reduce body temperature in growing feedlot steers and to determine the temperature threshold that sprinkler systems should be utilized to effectively reduce heat stress.

## METHODS

Animal care and experimental procedures were approved by the University of California (UC), Davis, Institutional Animal Care and Use Committee. Thirty-two Angus-cross steers (298 ± 15 SD days of age, 280 ± 38 kg initial body weight) from the UC Davis Sierra Herd were randomly selected, stratified by weight, and placed into eight treatment pens. Steers were halter broken over 14 d and provided a 5-d treatment acclimation period. All pens (30 m^2^) had existing metal shade structures that provided shade at a minimum of 7 m^2^ per animal. Pens were cleaned and bedded with straw once per week. Steers were fed a starter feedlot diet (2.88 Mcal ME/kg DM) twice per day at 0600 and 1600 hours according to UC Davis Feedlot protocol.

**Figure 1.**
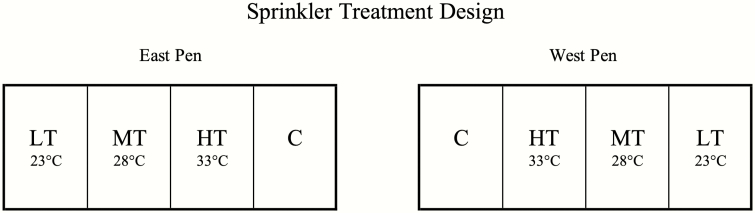
Sprinkler treatments included LT threshold (23 °C), MT threshold (28 °C), HT threshold (33 °C), and no sprinklers (C). Treatments were assigned to pens to accommodate water runoff, allocating one replicate on the east side of the feedlot and the other replicate on the west side.

Treatments included no sprinklers (C) and high (HT), moderate (MT), and low temperature (LT) threshold sprinkler systems activated at 33 °C (HT), 28 °C (MT), and 23 °C (LT), respectively. Treatments were assigned to the East and West replicate pens to avoid drainage issues as shown in Fig. 1. Sprinklers (Senniger Wobbler, 3/4, standard angle, #6 nozzle, Clermont, FL) were activated at corresponding temperatures at 30-min intervals for 5 min of groundwater delivery at 5.11 L/min. Each sprinkler head was fixed underneath the front of the shade structure in the middle of the pen, spraying 11 m in diameter.

Steer rectal temperature was measured in pen at 0800 (M), 1500 (A), and 1900 hours (E) with GLA Agricultural Electronics M700 Digital Thermometers (San Luis Obispo, CA) on Monday, Wednesday, and Friday each week for a total of 19 experimental days. Weather characteristics, including ambient temperature, wind speed, humidity, and solar radiation were collected daily from the KCADAVIS50 weather station at each measurement time. The minimum and maximum weather values for each day were also recorded.

Data analysis was performed using the LMER model in R Studio version 1.2.5033. Change in body temperature (ΔBT) from morning to afternoon and afternoon to evening was evaluated, with each individual animal serving as its own control. The first model tested the change in body temperature as the outcome variable with ambient temperature and treatment as fixed effects and individual animal as a random effect. The second model tested body temperature against fixed effects day and treatment with individual animal as a random effect. A one-way ANOVA and Tukey test with a 0.95 confidence level were also performed on both models for treatment comparison.

## RESULTS AND DISCUSSION

When ambient temperature rises, cattle are unable to expel heat load into the environment as efficiently, resulting in an increase in body temperature (Farooq et al., 2010). Feedlots can implement preventative measures to reduce animal adaptations to heat stress in an attempt to maintain thermal balance. Rise in ambient temperature can cause an increase in respiration rate, which results in up to 15% of heat loss by evaporative cooling (Finch, 1986), but additional methods of heat dissipation are necessary for complete thermal balance. Often, a decrease in appetite and feed intake will occur to reduce metabolic heat production (Blackshaw and Blackshaw, 1994).

**Figure 2.**
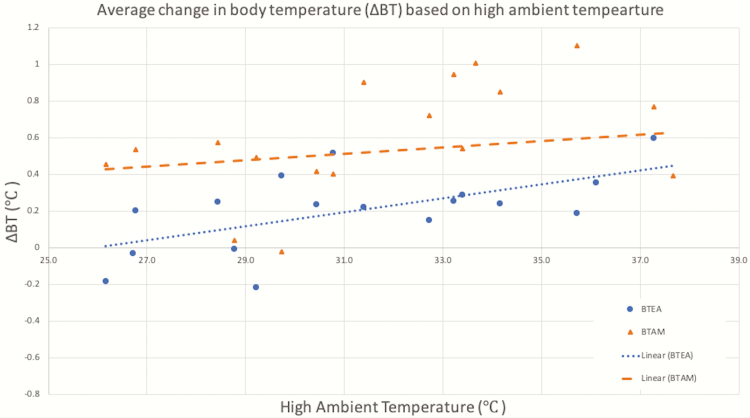
Average change in body temperature between the morning to afternoon readings (BTAM, triangles) and afternoon to evening readings (BTEA, circles) plotted against daily high ambient temperature conditions.

**Figure 3.**
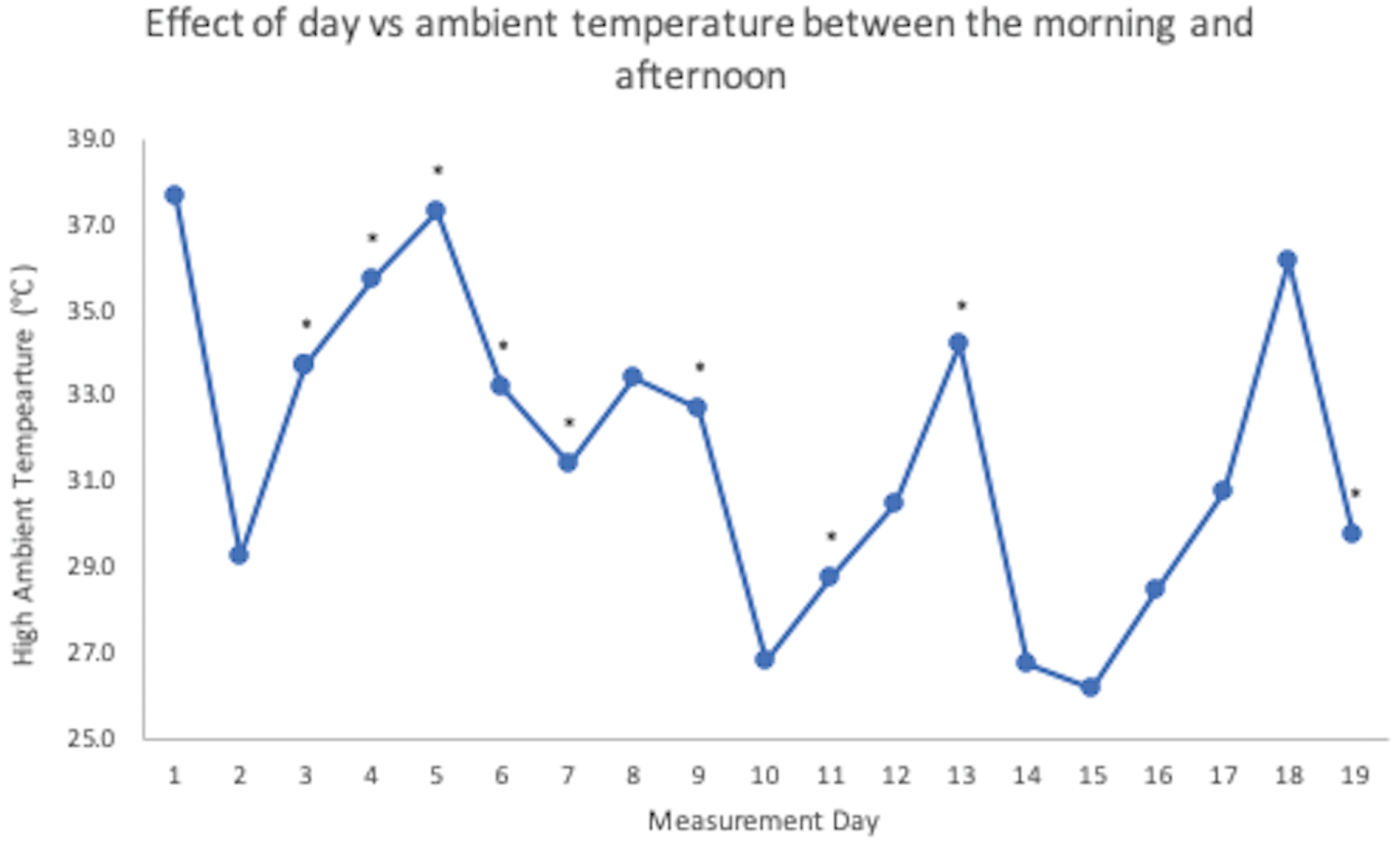
Daily high ambient temperatures for each day steer body temperatures were recorded. Asterisks above data represent days where the difference between morning and afternoon body temperatures were different (*P* < 0.05).

**Figure 4.**
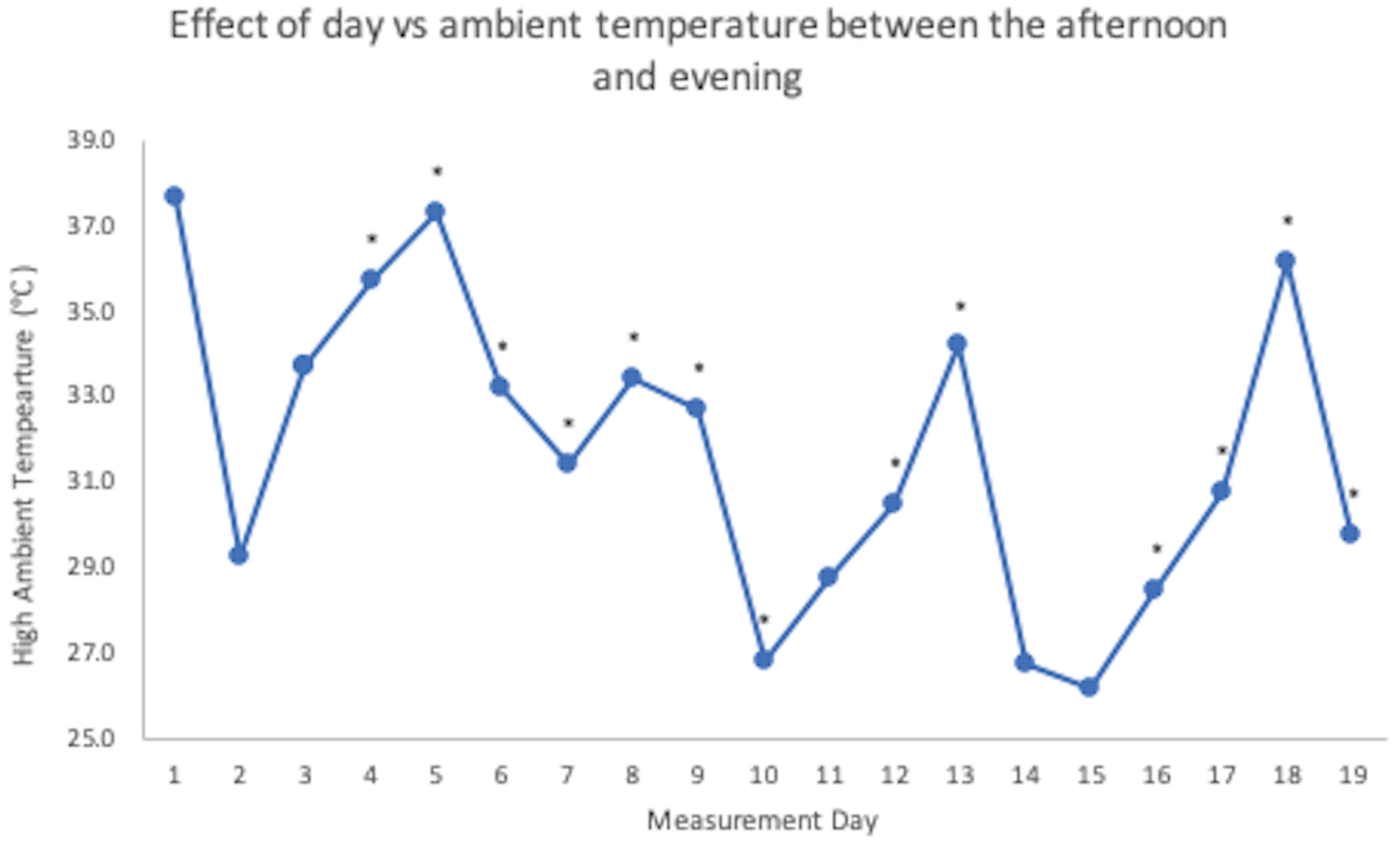
Daily high ambient temperatures for each day steer body temperatures were recorded. Asterisks above data represent days where the difference between afternoon and evening body temperatures were different (*P* < 0.05).

Heat stress can cause body temperature changes at as low as 26 °C and will increase in severity as ambient temperature surges (Berman et al., 1985). Daily high temperatures reached an average of 31 °C ± 3.6 SD on measurement, ranging from a low of 26.1 °C on day 15 and a high of 37.7 °C on day 1. As shown in Fig. 2, higher ambient temperatures generally caused a greater increase in ΔBT. The change in body temperature between the morning and afternoon was significantly affected by ambient temperature (*P* < 0.01). Day also significantly affected ΔBT (*P* < 0.01) between the morning and afternoon in nine experimental days as shown in Fig. 3. Between the afternoon and evening, day explained variation in ΔBT (*P* < 0.01) in 13 experimental days as shown in Fig. 4. Additional weather characteristics, such as wind speed, humidity, and solar radiation, are known to impact the level of heat stress (Silanikove, 2000) and should be considered for further analysis.

The timing and duration of sprinkler use can impact how effective the system is at reducing heat stress (Morrison et al., 1981). There was no treatment effect between the morning and afternoon measurements for HT (*P* = 0.6) compared to controls; however, a treatment effect was observed for MT (*P* = 0.02) and LT (*P* = 0.02). LT had the lowest average ΔBT, followed by MT, HT, and C, as predicted, though there was no significant difference between treatments (*P* = 0.07) as shown in Table 1. When averaged by day, ΔBT in the control group was significantly higher than LT (*P* = 0.03) and MT (*P* = 0.04).

**Table 1.** Average change in body temperature between the morning and afternoon and between the afternoon and evening measurements in sprinkler treatment groups

Sprinkler treatment^*a*^	Average change in body temperature (ΔBT)			
	ΔBT morning to afternoon, °C	*P*-value	ΔBT afternoon to evening, °C	*P*-value
Control (no sprinklers)	0.67		0.08	
HT threshold (33 °C)	0.64	0.68	0.22	0.07
MT threshold (28 °C)	0.53	0.11	0.22	0.07
LT threshold (23 °C)	0.52	0.07	0.30	<0.01

^*a*^Sprinklers were set to come on for 5 min every 30 min when ambient temperature exceeded the threshold.

High energy rations in feedlots increase heat load from metabolic activity, which contributes to one-third of the overall heat load experienced by cattle during high ambient temperatures (Finch, 1986). Between the afternoon and evening measurements, body temperature increased the most in the control group, followed by HT, MT, and LT. Afternoon feed delivery occurred at 1600 hours, following our afternoon temperature measurement. Cattle in the LT and MT pens that experienced a lower ΔBT from morning to afternoon may have consumed more feed when compared with the HT and C pens, therefore, resulting in an increased evening heat load. The LT group was significantly different (*P* < 0.01) from afternoon to evening as shown in Table 1. When averaged by day, the control group was significantly higher than the LT group (*P* < 0.01). The change in steer body temperature between afternoon and morning was affected by ambient temperature and, averaged across days, lowering the temperature threshold for sprinkling decreased the afternoon and evening body temperature increase in steers.

## IMPLICATIONS

For growing beef cattle, body temperature increases with greater ambient temperature in heat stress conditions. We show that sprinklers are most effective when applied at a lower temperature threshold, such as 23 °C. The LT threshold provided enough time for body temperature to remain lower for a longer period of time, reducing overall time in heat stress. After 23 °C, sprinkler efficacy is reduced as heat load is already rising in many of the animals. Sprinkler treatments can be an effective way to reduce body temperature; however, their effectiveness and practicality in large-scale application are still questioned.

Variation in response to heat stress was a prominent factor in the study, suggesting that many factors may contribute to the severity of heat stress on each individual animal. Understanding the extent to which different factors contribute to individual variation in heat stress response is essential. With further research, vulnerability scores may be developed to suggest which animals are at risk for severe heat stress, allowing producers to adapt their management strategies.
